# Enhancing chemical and physical stability of pharmaceuticals using freeze-thaw method: challenges and opportunities for process optimization through quality by design approach

**DOI:** 10.1186/s13036-023-00353-9

**Published:** 2023-05-23

**Authors:** Sergio A. Bernal-Chávez, Alejandra Romero-Montero, Héctor Hernández-Parra, Sheila I. Peña-Corona, María L. Del Prado-Audelo, Sergio Alcalá-Alcalá, Hernán Cortés, Lashyn Kiyekbayeva, Javad Sharifi-Rad, Gerardo Leyva-Gómez

**Affiliations:** 1 Departamento de Farmacia, Facultad de Química, Universidad Nacional Autónoma de México, Ciudad de México, 04510 Mexico; 2 Departamento de Farmacología, Centro de Investigación y de Estudios Avanzados del Instituto Politécnico Nacional, Ciudad de México, México; 3 Tecnologico de Monterrey, Escuela de Ingeniería y Ciencias, Campus Ciudad de México, Ciudad de México, Mexico; 4 Laboratorio de Tecnología Farmacéutica, Facultad de Farmacia, Universidad Autónoma del Estado de Morelos, Cuernavaca, Morelos 62209 México; 5 Laboratorio de Medicina Genómica, Departamento de Genómica, Instituto Nacional de Rehabilitación Luis Guillermo Ibarra Ibarra, Ciudad de Mexico, Mexico; 6 Department of Pharmaceutical Technology, Pharmaceutical School, Asfendiyarov Kazakh National Medical University, Almaty, Kazakhstan; 7 Faculties of Pharmacy, Kazakh-Russian Medical University, Public Health and Nursing, Almaty, Kazakhstan; 8 Facultad de Medicina, Universidad del Azuay, Cuenca, Ecuador

**Keywords:** Drug substances, Chemical stability, Physical stability, Hydrogels, Chemical properties, Pharmaceutical applications

## Abstract

The freeze-thaw (F/T) method is commonly employed during the processing and handling of drug substances to enhance their chemical and physical stability and obtain pharmaceutical applications such as hydrogels, emulsions, and nanosystems (e.g., supramolecular complexes of cyclodextrins and liposomes). Using F/T in manufacturing hydrogels successfully prevents the need for toxic cross-linking agents; moreover, their use promotes a concentrated product and better stability in emulsions. However, the use of F/T in these applications is limited by their characteristics (e.g., porosity, flexibility, swelling capacity, drug loading, and drug release capacity), which depend on the optimization of process conditions and the kind and ratio of polymers, temperature, time, and the number of cycles that involve high physical stress that could change properties associated to quality attributes. Therefore, is necessary the optimization of F/T conditions and variables. The current research regarding F/T is focused on enhancing the formulations, the process, and the use of this method in pharmaceutical, clinical, and biological areas. The present review aims to discuss different studies related to the impact and effects of the F/T process on the physical, mechanical, and chemical properties (porosity, swelling capacity) of diverse pharmaceutical applications with an emphasis on their formulation properties, the method and variables used, as well as challenges and opportunities in developing. Finally, we review the experimental approach for choosing the standard variables studied in the F/T method applying the systematic methodology of quality by design.

## Introduction

The cross-linking of polymers alters their physical properties, allowing different applications at the macro-, micro-, and even nanomolecular levels. The linking of polymer chains through chemical bonds or physical interactions has enabled the development of numerous functional materials, including thermosets, rubbers, and hydrogels [1]. Methods for chemical cross-linking of polymers are classified into cross-linking during polymerization and subsequent cross-linking of polymer chains and are generally promoted by the addition of catalysts and/or cross-linking agents that form covalent bonds. Physical cross-linking is done using interactions other than covalent bonding, such as hydrogen bonding or ionic interaction, and they can reversibly dissociate and recombine under specific stimuli such as heating/cooling [2]. Although chemical cross-linking is a very versatile method to create hydrogels with good mechanical stability, the cross-linking agents are often toxic compounds and cause unwanted reactions to the hydrogel matrix’s bioactive substances. Such adverse effects can be avoided using cross-linked gels by physical methods [3].

The freeze-thaw (F/T) method is applied in the pharmaceutical field to produce polymeric matrices through consecutive F/T cycles. During freezing, the solvent crystallizes, concentrating the polymer chains in solvent regions and promoting zones of physical unions that remain when thawed. In this way, the self-assembly of polymers is promoted, mainly through hydrogen bonding interactions, and without the requirement of chemical cross-linking agents [4]. Therefore, the F/T method is simple, environmentally friendly, and easily scalable.

The pharmaceutical applications of F/T include the synthesis of hydrogels, emulsions, nanosystems, and films, with the primary objective of transporting drugs. Although it also has applications in tissue engineering, agriculture, cosmetics, wastewater treatment, and others [5]. A material widely used in this method is poly(vinyl alcohol) (PVA), a synthetic polymer with abundant hydroxyl groups, low toxicity, biodegradable, cytocompatible, and low price. Its applications are limited by its intrinsic characteristics, such as porosity, swelling capacity, flexibility, and drug loading, which depend on the type and proportion of polymers and the F/T conditions. However, PVA is also biocompatible with other biopolymers with different characteristics, such as cellulose, chitosan, gelatin, casein, and others, which enables various applications to the matrix resulting from the coupling [5–7]. The aim of the study is to review and discuss different studies related to the impact and effects of the freeze-thaw (F/T) process on the physical, mechanical, and chemical properties of diverse pharmaceutical applications, with an emphasis on their formulation properties, the method and variables used, as well as challenges and opportunities in developing. The study aims to provide an understanding of how the F/T method is employed in the processing and handling of polymers to enhance their chemical and physical stability, improve formulation stability, and increase drug concentration in pharmaceutical applications. The study also aims to highlight the challenges and opportunities in optimizing the F/T conditions and variables to ensure safety and efficacy in the use of this method. Finally, the study aims to explore the experimental approach for choosing the standard variables studied in the F/T method applying the systematic methodology of quality by design.

## Fundaments of freeze/thaw

### Background

Seminal investigations on the F/T method were first reported by Peppas in 1975 [8], resulting from experiments focused on unveiling the potential influence of the crystalline state of polymers on the properties of diverse crystalline materials. Peppas observed by infrared spectroscopy that the heating processes caused a change in the intensity of the band of infrared spectroscopy at 1141 cm^−1^, a normal mode associated with the symmetrical C-C stretch in PVA [9]. This observation allowed him to elucidate that heating the polymer directly modified its degree of crystallinity. It was later demonstrated that the crystalline fraction of PVA does not allow water permeation in the resulting materials, while amorphous and semi-crystalline phases are susceptible to swelling [10]. Although the method was developed initially to perform structural observations of PVA under different conditions and to avoid heating, this technique allowed tuning the properties of highly organized polymeric materials by applying the F/T under specific times and temperatures. Thus offering a convenient post-synthetic method to obtain different crystallinities and pore sizes for the same polymer [11, 12].

These studies on PVA were contemporary to independent research directed at finding new chemical cross-linking processes that would yield materials with robust physicochemical properties without using toxic agents [13]. Thus, Peppas’ theory of controlling the physical properties of materials through the control of crystalline states was eventually extrapolated to other polymers and blends of polymers, leading to the popularization of the F/T technique. The implications of various modifications in the method conditions (cycle lengths, temperatures, and polymer concentration) have been reported for different systems. Here we will emphasize the use of this technique in elaborating materials with pharmaceutical applications. Nevertheless, F/T is used mainly to reinforce the mechanical properties of the materials to be obtained due to the crystallization process [14, 15].

### The scientifical and technical basis

**Fig. 1:**
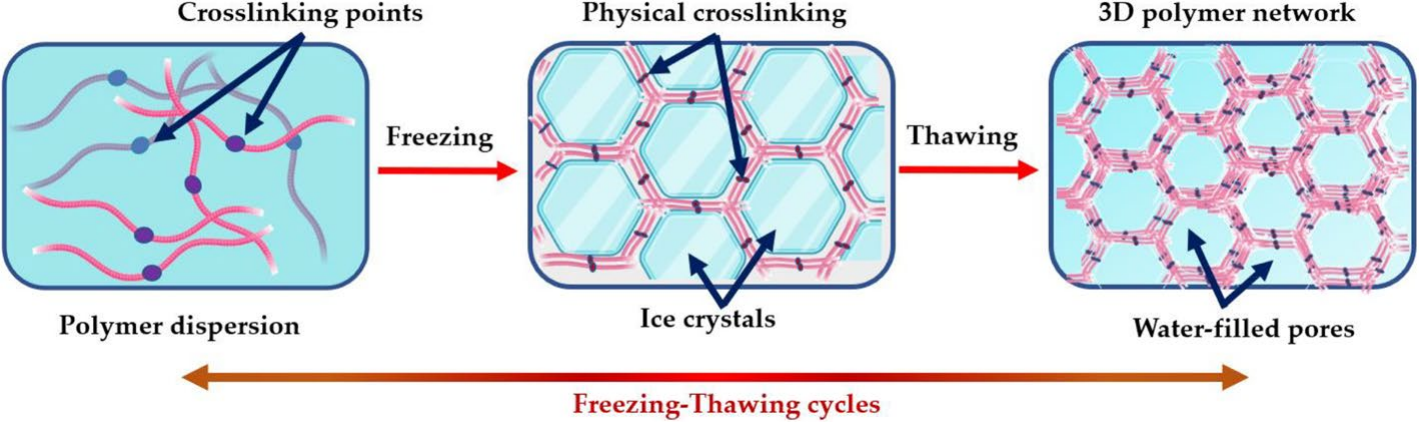
Schematic representation of the freeze-thaw process

The F/T process, illustrated in Fig. 1, occurs due to freezing the water contained within the polymer dispersion during exposure to low temperatures (-35 °C to -20 °C), expelling the polymer chains assembled in high-concentration regions. Close contact among these chains promotes the crystallization process and the formation of hydrogen bonds that act as cross-linking points [16, 17]. While the F/T cycles continue, the residual molecular chains are successively included in the concentrated bonding zones. These interactions remain intact during thawing, creating a non-degradable three-dimensional polymer network [18].

According to the literature, the initial polymer concentration and the number of F/T cycles influence the crystallinity, compressive and tensile modulus, and size and morphology of the resulting pores [19]. Initially, increasing the polymer concentration results in higher nascent crystallinity and added stability after swelling. However, a too-high crystallinity damages elasticity and makes the gels more brittle [20]. Consequently, modulating the initial concentration is also a determining factor. Although this methodology was primarily utilized for hydrogels, its usefulness with other polar solvents has also been demonstrated [21].

**Fig. 2:**
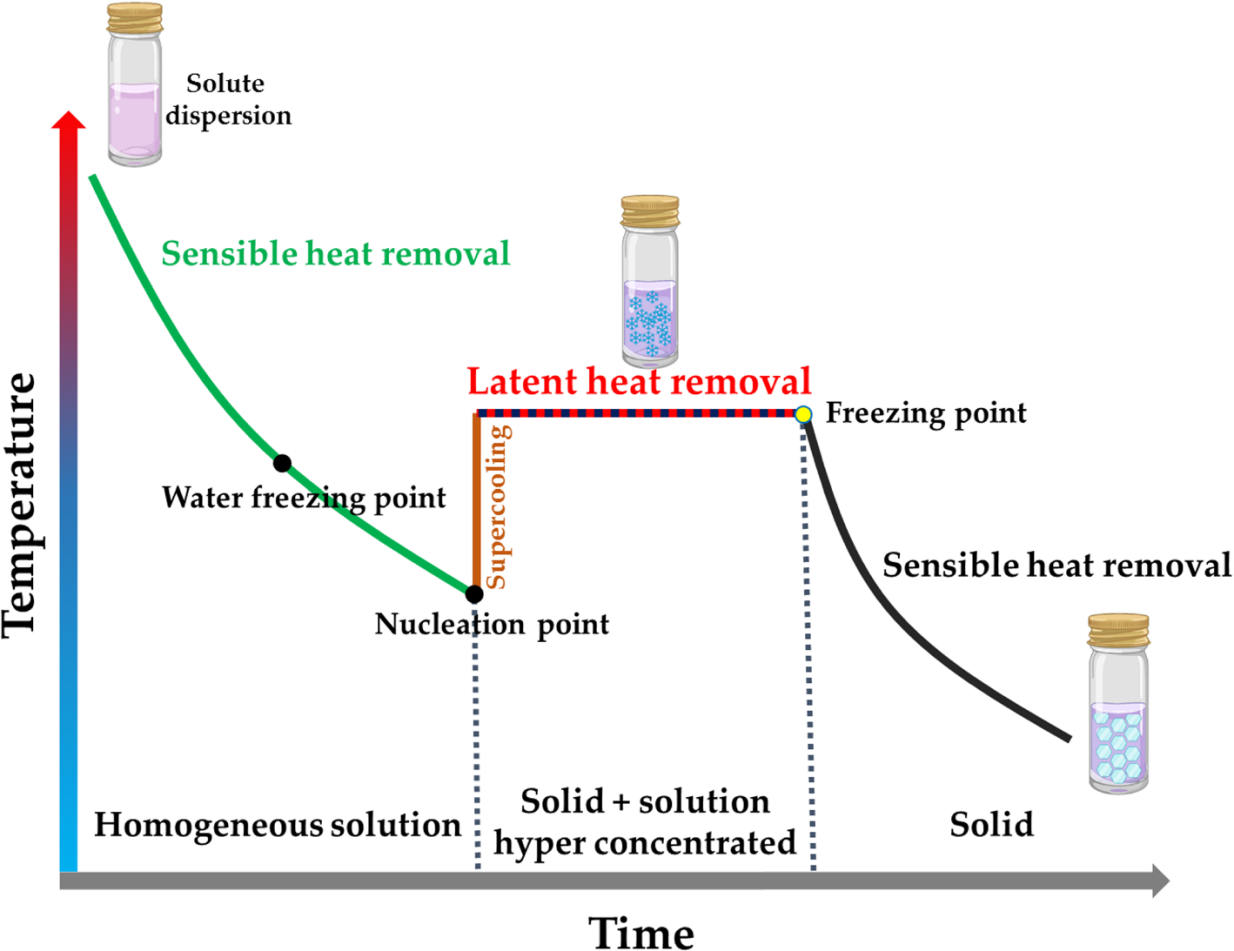
Typical freeze-thaw time-temperature curve

The freezing process involves different molecular stages, as shown in Fig. 2; this phase transition is divided into four successive stages: supersaturation/supercooling, nucleation, crystal growth, and recrystallization [22, 23]. The formation of water crystals is the most critical step, as it will determine the morphology and stability of the three-dimensional pore structure of the resulting material. Their formation occurs when the liquid phase exceeds equilibrium conditions and is supersaturated or supercooled; this is easily achieved by reducing the temperature below the melting point and/or by increasing the solute concentration in the solution above the saturation point [23].

In the case of polymeric solutions, the polymer itself serves as the starting point for the nucleation phenomenon by acting as a catalyst; nucleation is dependent on the system components and their concentration, as well as the holding temperature, cooling rate, and shear forces. At this stage, the temperature remains constant because the molecules release heat when they change their organization from a liquid to a crystalline structure; this represents the latent heat of crystallization [24, 25]. The crystal growth rate depends on the magnitude of saturation or cooling of the system, i.e., the rate at which latent heat is removed from the liquid-solid boundary [26]. The water crystals continue growing until the thermodynamic equilibrium state is reached. Simultaneously, the polymer (initially dissolved in the aqueous phase) moves to the solid-liquid boundary, increasing its concentration in the aqueous phase [27, 28].

The rising polymer concentration leads to a progressive melting point reduction during the whole crystallization phase. In the end, the system is formed by two separate phases: the crystals and the hyper-concentrated non-frozen solution of the polymer [29]. The composition of the solution and its physicochemical properties can be drastically different from the initial ones, including potential changes in pH, ionic strengths, osmotic pressure, or viscosity, depending on the nature of the polymer [30]. Hydrogen bond formation occurs with this variation in properties stabilizing the gel, emulsion, or nanoparticles obtained [31].

The size and number of crystals that subsequently determine the pores’ morphology depend on the nucleation and growth rate of the crystals [32]. The faster the cooling rate, the higher the nucleation rate becomes compared to the crystal growth rate, yielding increased nucleation points and giving rise to many tiny crystals [32]. On the contrary, if the cooling ramp is slow, the nucleation rate is lower than the growth rate, giving rise to a few large crystals. Subsequent recrystallization occurs by splitting or bonding the preformed crystals due to changes in the temperature or humidity of the system. Then the materials must be kept in temperature-controlled systems, usually below -18 °C [33].

Several strategies have been developed to maintain the stability of the three-dimensional structure, such as modulating the chemical composition of the mixture, precisely controlling cooling times and rates, and employing additives that serve as cryoprotectants such as polysaccharides, polyols, and proteins [22]. Although it has been exposed that the thawing phase also influences the final structure of the materials, it is evident that the freezing step is decisive. Since very few studies describe the influence of the thawing process, this is an opportunity to improve the manufacturing of materials obtained by this technique.

### Method conditions

In the F/T process, the most critical parameters are (i) the number of cycles and their duration, which vary depending on the targeted material and its chemical composition, but generally range between 3 and 10 cycles; (ii) the freezing rate: as already mentioned, the freezing speed influences the shape and size of the water crystals and consequently the pore size and the formation of cross-linking; (iii) the thawing rate must be controlled to avoid overheating and conducted below the melting point so as not to melt the polymer network; (iv) the medium in which the process is carried out should help to maintain hydration and avoiding osmotic stress while being compatible with the raw materials by not intervening in their structure and properties, and finally (v) the materials must be stored at a temperature below the freezing point to help their preservation. These parameters and their influence on the final materials are listed in Table 1.

**Table 1:** Traditional method conditions

**Factor**	**Effect**	**Specification**	**Reference**
Freezing rate and temperature	It influences mechanical stress, pores size, and sharp edges	12 h, room temperature until -80° C	[11, 12]
Cycles (time and length)	It affects thermal stability and crystallinity degree	Between 3 and 10	[34–36]
Thawing rate and temperature	It impacts three-dimensional network stability	-80 °C to 4 °C, in 20 min	[31]
Medium	It determines suitable physicochemical properties	Water, ethanol, methanol, DMSO, acetone	[10, 37]
Solute concentration	It exerts a direct influence on the rate of transition phases	Saturated solution	[25, 38]
Additives	They provide structure stabilization	Polysaccharides, polyols, and proteins	[39, 40]
Solute nature	It influences mechanical properties and chemical functionality	Polymers, oils, proteins, and nanoparticles	[16, 22]
pH changes	It affects swelling and mechanical properties	non-ionic	[30, 38, 41]

### Special considerations of the freeze-thaw method for emulsions

Emulsions are two-phase systems composed of two immiscible liquids where one phase is dispersed as globules in the other continuous phase. Due to their thermodynamic instability, emulsifying agents are added to ensure stability [42]. These agents form a thin layer around the globules of the dispersed phase to prevent coalescence and separation. Pharmaceutical emulsions consist of mixtures of an aqueous phase and a phase comprising oils or waxes, serving as carriers for oil-soluble drugs. F/T processes can cause destabilization in emulsions, leading to coalescence, flocculation, breaking, and creaming [43]. To mitigate these effects, it is necessary to avoid solution saturation during freezing, which can dehydrate the interfacial surfactants and alter their properties. For example if salts are added in high concentrations can eliminate any dielectric repulsion between droplets and promote their merging [44, 45]. During freezing, the volume fraction of the dispersed phase increases, and the droplets are subjected to stress from the expanding ice phase. If emulsion droplets are trapped and concentrated between growing ice crystals, their stability relies on the integrity of the membrane separating them. The type of lipid can influence the transmission of forces produced by ice to the membrane, highlighting its role in emulsion stability [46].

### Special considerations of the freeze-thaw method for nanosystems

F/T can cause stress to the colloidal suspension of nanoparticles, particularly during the freezing and dehydration stages. The cryoconcentrated phase that forms during freezing can induce aggregation and melting of the nanoparticles, while ice crystallization can cause mechanical stress and destabilization [47, 48]. To protect these fragile systems, special excipients such as cryoprotectants and lipoprotectants are added prior to F/T. These excipients not only protect the nanoparticles from freezing and drying stress but also increase their stability during storage [49]. The F/T of nanoparticles is a complex process that requires careful consideration of both the formulation and process conditions. The success of F/T is influenced by many factors, such as the composition of the nanoparticles (e.g., type of polymer, concentration and type of surfactant, concentration and type of cryoprotectants and lipoprotectants, interaction between cryoprotectants and nanoparticles, surface modification of nanoparticles), and the conditions applied during F/T. Factors such as freezing rate, annealing, pressure, temperature, and duration of each process step can affect the stability of the nanoparticles during and after F/T [50, 51]. Thorough investigation and optimization of these parameters are necessary to achieve successful F/T of nanoparticles.

### Advantages and disadvantages

Among the main advantages of the F/T method are the simplicity and efficiency of the process because it does not involve sophisticated laboratory equipment and is easily scalable and highly reproducible. These characteristics make it economically viable, which is fundamental to guarantee its widespread use in pharmaceutical and medical applications [52]. The resulting materials are homogeneous, an essential characteristic for developing appropriate mechanical properties and the biological performance for which they were intended [53]. The method allows tailoring the polymer to obtain desired properties such as swelling, degradation rate, mechanical resistance, and porosity of the final product to reach specific pharmaceutical applications [54].

However, there are still several areas of opportunity to improve the applicability of this process for obtaining pharmaceutical materials. Some of its disadvantages come from the difficulty of correctly homogenizing the initial polymeric solution, which produces areas with a greater amount of water that will cause an inhomogeneous pore formation resulting in compromised mechanical properties by points of failure or cracks [55]. In this regard, if the water crystals formation is uncontrolled, they can form abrupt sharp edges that can break the polymeric network irreversibly, damaging the mechanical properties [56, 57]. The rate and duration of the cycles can also reduce the stability of the material because they directly affect the physicochemical properties of the material if they are not adequately controlled [58, 59]. All these difficulties in the pharmaceutical field make it challenging to tailor polymers by the F/T technique without degrading the molecules added as active ingredients and other biological components such as cells, enzymes, or genes that could be part of the formulation [55, 60].

## Impact and effects on physical and chemical properties

It has been reported that the hydrogels’ structure and characteristics, like mechanical properties, stability, or porosity, depend on the effect of specific parameters such as the polymer’s molecular weight, concentration, and F/T conditions (e.g., number of cycles and temperature). Here, we discuss the effect of the F/T process and the number of cycles on the physicochemical properties of hydrogels.

### Mechanical properties

The mechanical properties of materials for tissue engineering or pharmaceutical purposes play a crucial role in the desired application. The elasticity module values vary in function of the organ of interest and could modify the release rate of drugs or bioactive molecules in delivery platforms. In this context, some researchers have demonstrated that the number of F/T cycles and the temperature highly affect the mechanical properties [58, 61]. Specifically, Lozinsky et al. experiments revealed that rapid freezing in the polymer solution triggers the cracking of the PVA hydrogel [61]. Furthermore, the authors reported that hydrogels exposed to several F/T cycles (about 45 cycles) increased the number of crystallinity domains, increasing the mechanical strength and decreasing the swelling ratio.

Similarly, Gupta et al. reported that the percentage of crystallinity varies depending on the number of F/T cycles; however, they found that 15 and 45 cycles presented the same crystallinity percentage at 16% of PVA [58]. Thus, the increase in PVA concentration also increased the formation of stable crystalline regions, which benefit chain folding. Interestingly, this behavior could be suppressed at lower F/T cycles inhibiting the chain folding. The repeat F/T cycles promote the chains’ polymer alignment in the liquid microphase (unfrozen), triggering the formation of intermolecular aggregations.

On the other hand, slow thawing combinate with a slow cooling rate produces a higher modulus of elasticity in hydrogels [62]. Kim et al. analyzed the stiffness of PVA hydrogels created through the F/T technique to evaluate this parameter’s effect on stem cell differentiation [63]. The authors developed a PVA hydrogel frozen gradually in the solution along the longitudinal direction from the bottom to the top using liquid nitrogen (LN_2_). Besides other remarkable results, the authors reported that the crystallinity of PVA hydrogel increased with lower freezing temperatures and longer freezing times, providing enhanced mechanical properties. Furthermore, the compressive modulus varied from 1.47 ± 0.32 kPa to 23.99 ± 3.60 kPa, from top to bottom. This stiffness gradient is intimately related to the crystallinity gradient created by the freezing time and temperature differences during the freezing process.

Similarly, a glycol-chitosan-catechol hydrogel was prepared by F/T under varying procedure conditions [19]. The authors evaluated the catechol cross-linking chemistry at room temperature and cryo-conditions (in the F/T process) and reported a dramatic increment in storage modulus value in the F/T cryogels (3–sixfold) compared to the room-temperature hydrogels. Furthermore, their results demonstrated that the frozen state accelerated hydrogel gelation.

In 2017, Hong obtained hydrogels of PVA and acid tannic (TA) by three cycles of F/T conditions, observing a loss of transparency compared with hydrogels prepared by other methods. This characteristic is related to the binding of molecules derived from the F/T process [64]. The authors also reported that the hydrogel hardness increased as TA augmented. Similarly, using polysaccharides (such as xanthan gum or salecan) combined with PVA allowed the obtention of hydrogels with a wide range of applications. However, these polysaccharides decreased the compressive strength and the compressive modulus [65, 66].

### Porosity

Well-organized architectures, large surface areas, and tunable pore sizes characterize porous materials. These properties could enhance the success of biomaterials due to the necessity of porosity for the interaction between cells and biological environments [67–69]. In the case of controlled drug delivery structures, incrementing the surface area gives more reactive sites per material surface unit, which permits obtaining a more efficient system [70, 71].

The porosity characteristics (such as pore size and distributions) could be controlled by F/T conditions (cooling rate, cycle times, cryoconcentration, etc.) [19, 34, 72]. For instance, incrementing polymer concentrations results in smaller pores or thicker walls [62, 73]. In the freezing procedure, the ice crystals act as a porogen in the hydrogel formation, providing porosity [63]. Furthermore, the abundant hydroxyl groups in polymers employed for this purpose, such as PVA, chitin, hyaluronan, and hemicellulose, stimulate the physical cross-linking derived from the process [59, 74]. On the other hand, the thawing process promotes the interactions between the remaining polymers, provoking the formation of networks in the hydrogel [62, 75]. Additionally, the number of cycles could also impact the morphology due to an increment in the cycles leading to more defined pores.

Recently, Figueroa-Pizano et al. reported that chitosan-PVA hydrogels treated at lower freezing temperatures (such as -80 °C) presented smaller pores than those treated at higher temperatures (-4 °C) [34]. Their results suggested that the solvents employed and the polymer concentration are parameters that could modify the pore structure and porosity percentage, e.g., higher chitosan concentrations produce a more porous network and broader pores. The increase in the number of F/T cycles causes a high hydrogen bonding formation between PVA polymer chains and chitosan, which enhances the cross-linking degree in the hydrogels and crystallization of PVA chains [59].

Some authors have prepared hydrogels using the F/T method and other cross-linking techniques, such as photo-cross-linking and chemical cross-linking, to evaluate the changes in materials’ properties. The material’s structure could be reduced by increasing the photo- and ionic-cross-linking derived from the presence of components such as kappa-carrageenan [76].

### Swelling capacity

The evaluation of water content in hydrogels for pharmaceutical applications is a requirement, especially for those that serve as wound dressings, because they provide a local moist environment and enhance the nutrients, drugs, or protein permeation into the target site [77, 78]. The hydrogel swelling is directly related to porosity and pore size. The polymer’s relation and solvent concentration are determinants in the swelling ability, besides the properties of the polymer (hydrophilicity). For example, it was reported that an increment of TA concentration in a TA/PVA hydrogel decreased swelling properties, which is also related to microporosity [64]. Similarly, higher concentrations of hydroxyapatite trigger a lower equilibrium swelling ratio in hydrogels developed for bone regeneration due to the decrement in the amorphous region volume [62, 79].

Some researchers showed that the swelling capacity decreases when the number of F/T cycles increases. This behavior is attributed to the fact that the increment in cycles increases the crystallinity in gels, restricting the movement of the polymer chains and suppressing the swelling ratio. Then, the number of F/T cycles and the orientation of these processes could change the swelling kinetics of materials.

In 2018, a notable study evaluated the influence effect of stretching within the formation of F/T cycles on the hydrogel properties [74]. The orientation allows the polymer chains to form uniaxial alignments, which increases the swelling capacity compared to those without orientation. This behavior could be interesting for drug delivery science due to its direct impacts on the hydrogel release capacity; higher swelling capability allows the quick release of the drug or molecule inside the hydrogel.

The different properties and characteristics obtained in hydrogels by modifying parameters in the F//T process allow for obtaining a wide range of applications for these materials, making this technique versatile and effective in pharmaceutical applications.

### Structural implications of the freeze-thaw process in emulsions and nanosystems

During freezing, the formation of ice crystals can cause the nanoparticles or oils drops in emulsions to aggregate or agglomerate, which can lead to an increase in particle size. Upon thawing of the sample, the ice crystals melt and the resulting liquid can cause the nanoparticles/drops to re-disperse [80, 81]. However, if aggregation or agglomeration during freezing is significant, the nanoparticles may not completely re-disperse, resulting in a permanent increase in particle size [49].

On the other hand, freeze-thaw cycles can also induce changes in the morphology and size distribution of nanoparticles, especially for some types of polymers and surfactants. For example, freeze-thawing can induce the formation of new crystalline structures or modify the morphology of nanoparticles, leading to changes in particle size [82, 83].

It is important to note that the effect of freeze-thaw cycles on nanoparticle size can depend on several factors, such as the specific properties of the nanoparticles and the freeze-thaw conditions, such as freezing rate and temperature. Therefore, the impact of freeze-thaw cycles on nanoparticle size must be carefully evaluated for each specific system [84].

## Pharmaceutical applications of freeze-thaw

### Hydrogels

Hydrogels are three-dimensional polymers produced by chemical and/or physical reactions that cause “tie points” formed by covalent or ionic bonds, strong entanglements, crystallites, or hydrogen bonds [30]. The effects of gel properties and swelling deterioration are minor in non-ionic hydrogels [30]. The F/T method in hydrogels has been used as a physical cross-linking strategy, which makes it possible to replace chemical cross-linking agents since the latter could cause adverse effects, such as undesirable reactions with other components (if any). The solutions of polymers, such as PVA, become gels upon repetitive F/T cycles due to phase separation and crystallization [30]. This sol-gel transition produces a thermoreversible gel, a matrix of a physically cross-linked polymer containing uncrosslinked polymer, and water [52]. Hassan and Peppas [85] studied freeze-thawed PVA hydrogels’ structure and morphology by repeating cycles of 8 h of freezing at -20 °C and 4 h of thawing at 25 °C. The authors found that an increase in the PVA molecular weight resulted in crystals of higher lamellar thickness and a broadening of the crystal size distribution due to the rise in PVA chain length. The crystallization was more pronounced for more loosely cross-linked samples. On the other side, PVA has also been mixed with other polymers. For instance, Figueroa-Pizano et al. developed a PVA-chitosan hydrogel by the F/T method [34]. The presence of chitosan content increases pore size based on SEM micrographs and the swelling ratio. The increase in the number of F/T cycles causes the hydrogen bonding between PVA polymer chains and chitosan, which enhances the cross-linking degree in the hydrogels and crystallization of PVA chains, as was observed by Moradi et al. [86]. The F/T method has also been used as a strategy for preparing aerogels. Huang et al. [87] prepared functional aerogels from cellulose nanofibrils and carbon nanotubes, constructing aerogel pore walls with suitable mechanical properties by cycling F/T to cross-linking the cellulose nanofibrils and carbon nanotubes. The aerogels exhibited tunable densities, high specific surface area, and appropriate conductivity, and they are easily recyclable due to the absence of chemical cross-linking. The aerogel was frozen at -45 ºC for 30 min, thawed at 25 ºC for 30 min, and cycled this process thrice.

Other polymers that have been used for the development of hydrogels based on the F/T method, alone or in combination with PVA, are carboxymethyl cellulose [88], locust bean gum [89], kappa-carrageenan [90], among others.

The evaluation of the syneresis of hydrogels is another area where the F/T has been applied. The syneresis test is a primary method for evaluating the ability of hydrogels to resist undesirable physical changes upon repeated F/T. Overall, the syneresis values of the gel samples increased with the number of F/T cycles, especially during the first cycles. Wang et al. [91] found that the syneresis values for the kappa–carrageenan gels incorporating a composite of cinnamon essential oil-hydroxypropyl–β–cyclodextrin were significantly lower than those for the pure kappa-carrageenan gel during the five F/T cycles. The results indicated that the composite significantly enhanced the F/T stability of the kappa–carrageenan gel because it binds to a portion of the free water present in the pure gel, hindering its transfer from the gel network and the formation of ice crystals during freezing. Consequently, less water was released from the gel network upon thawing.

### Emulsions

F/T is utilized in emulsions for the production of highly concentrated emulsions, stability evaluation, morphology characterization, mass transfer within emulsions, formation and dissociation of hydrate in emulsion systems, preparation of composite fiber with porous shell structure, thermal storage material, and demulsification of W/O emulsions [92].

A study by Rojas et al. [93] demonstrated that, in double emulsions W1/O/W2, freezing the oil phase (O) preserves stability. At the same time, subsequent thawing triggers the coalescence of the droplets of the internal aqueous phase (W1) with the external aqueous phase (W2), termed external coalescence. This study obtained temperature-sensitive bulk W1/O/W2 double emulsions that potentially allow denaturation components such as chemical penetration enhancers may be stored within the oil and continuous (W2) phases without modification of the composition in the interior droplets (W1), chosen to minimize protein instability during storage [94].

In the same way, most simple O/W emulsions are destabilized after F/T because of the crystallization of the oil and water in the respective phases through coalescence or partial coalescence. Ice crystals can destabilize emulsions by flocculating oil droplets, increasing ionic strength, and changing the pH in the unfrozen aqueous phase. Fat crystals can penetrate neighboring oil droplets during freezing, which causes partial coalescence in the O/W emulsion that collapses to form larger droplets, which appear in the separated oil when the emulsions are thawed [95]. These emulsion stability studies were analyzed by Cornacchia and Roos [96] using DSC, gravitational separation, and particle size analysis during four F/T cycles. The emulsions were stable when only lipid crystallization occurred. DSC data indicated that lipid crystallization in emulsions containing hydrogenated palm kernel oil caused destabilization at low sucrose concentrations before water crystallization. The emulsions were stable if the dispersed oil phase crystallized after the dispersing water phase (such as emulsions containing sunflower oil), exhibiting that during F/T cycling, the crystallization temperature of the dispersed lipid phase plays a crucial role in the stability of oil-in-water emulsions stabilized by dairy proteins.

### Nanosystems

Freezing is the critical stress for products stored as frozen liquids or lyophilized substances [97]. The F/T method has been used to evaluate the stability of different nanosystems. For example, nanoparticles based on siRNA often suffer from aggregation and loss of function during storage [98]. An alternative method for preserving the dispersibility of nanoparticles is to freeze the suspension. However, freezing a suspension of nanoparticles is also accompanied by nanoparticles concentration and their consequent aggregation. Furthermore, thawing a frozen suspension could be unfavorable for preserving nanoparticle dispersity. These aggregation pathways due to freezing can be interrupted by adding adequate and appropriate substances to the suspension [99]. For example, PVP has been reported to exert marked inhibition of aggregation of silica nanoparticles during F/T, where the freezing was in LN_2_, and the thawing was at 60 ºC for 30 min [100]. In the case of aluminum oxide nanoparticles, the addition of potassium phosphate, sodium citrate buffers, or stabilizers such as PVA or gelatin A/B reduces the increased nanoparticle size after being subjected to 3 cycles of F/T, frozen at -1 ºC/min to -50 ºC and thawed at 1 ºC/min to 10 ºC with 90 min in both stages. Therefore, the authors identified that the fundamental formulation principles to preserve inorganic nanoparticles upon freezing are maintaining the pH during freezing and adding a suitable stabilizer [97].

Lee et al. [101] developed polycaprolactone nanocapsules (with eugenol as a model drug), which were subjected to F/T cycles in the presence and absence of cyclodextrins. The nanocapsules without β-cyclodextrin were remarkably aggregated at all freezing temperatures; however, no significant particle aggregation occurred in the nanocapsules with cyclodextrins. Therefore, the β-cyclodextrin was considered a cryoprotectant resulting from the retarded particle aggregation during the freezing process.

It has been observed that in gold nanoparticles, a higher thawing temperature (from 4 ºC to 60 ºC) resulted in 10–20% better dispersibility of the nanoparticles, whereas the freezing temperature (-20 ºC in the freezer or -197 ºC in LN_2_) had no significant effect. Correspondingly, the increase in aggregation index value due to F/T was generally more significant than that due to freeze-drying [99]. However, the F/T method is short and quick compared to freeze-drying and hence can be employed as a pre-test to screen the type and concentration of cryoprotectants used in freeze-drying. F/T study is based on the principle that if an excipient cannot protect the nanoparticles during the first step of freezing in the lyophilization, it is not likely to be an effective cryoprotectant [102].

In this context, Noga et al. [103] studied the effect of F/T (-50 ºC/10 ºC) and freeze-drying on the particle size of hydroxyethyl starch (HES)- and PEG-coated polyplexes. PEG-coated polyplexes exhibited a significant increase in particle size up to 3000 nm after 3 F/T cycles. Meanwhile, HES-decorated polyplexes revealed higher stability, as the increase in particle size is lower (only up to 500 nm after 3 F/T cycles). The addition of cryoprotective excipients markedly reduced the tendency toward particle clustering. All lyophilized polyplexes exposed a good cake appearance after the freeze-drying process with short reconstitution times in water (< 3 s).

Likewise, products based on proteins can be exposed to the stress of F/T transition during production, purification, storage, transport, and delivery to patients. It can cause the formation of subvisible particles. The evaluation of the aggregation of nanoparticles after being subjected to F/T cycles should be considered during their development since it appears to play a role in microparticle formation in protein formulations [104].

On the other hand, nanoparticle coating can also be affected by F/T cycles. For example, when gold nanoparticles are protected with a lipid bilayer and subsequently treated by F/T (-20 ºC at least 1 h and room temperature with shaking or sonicating, respectively), the F/T process induces fusion or fission of lipid bilayers tethered on the nanoparticles. UV-VIS spectra and transmission electron microscopy experiments revealed that the disruption of lipid bilayer structures on the nanoparticles led to the fusion or aggregation of nanoparticles [105].

In addition to being a method utilized to evaluate the effect on the aggregation, stability, or disruption of lipid coatings on nanoparticles, the F/T process has also been employed to fabricate nanosystems, especially in supramolecular complexes of cyclodextrins and liposomes. Maestrelli et al. [106] investigated cyclodextrins complexation and entrapment in liposomes to develop an effective topical formulation of ketoprofen. The encapsulation efficiency increased with increasing the cyclodextrins complex concentration. However, an opposite behavior was observed for frozen and thawed liposomes, probably due to the freezing phase required by such a preparation method, which reduced the complex solubility due to the drug precipitation arising during the freezing cycle. Likewise, these liposomes exhibited a less regular morphology due to the “traumatic” preparation method, as indicated by the researchers. The authors state that the cycles of F/T give rise to the rupture of the phospholipid bilayer, which can be reconstituted in an untidy lamellar structure.

Conversely, Tao et al. [107] prepared inclusion complexes of thymol and thyme oil with β-cyclodextrins using F/T and kneading processes. The authors reported that the F/T procedure produced higher entrapment efficiency than kneading, indicating its potent efficacy. Zhao and Lu [108] also observed this encapsulation efficiency increase when encapsulating recombinant human growth hormone in liposomes using a modified F/T process where three to four cycles of F/T generated higher encapsulation percentages.

Finally, Elorza et al. [109] prepared 5-fluorouracil-loaded liposomes by reverse-phase evaporation and F/T extrusion in LN_2_ and warm water (50 °C). The authors found that liposomes prepared by reverse-phase evaporation were more heterogeneous in vesicle size than those obtained by F/T extrusion. Even the reproducibility of the results was adequate for liposomes obtained by F/T and somewhat poorer for reverse-phase liposomes. Moreover, the phospholipid recoveries were above 90% and around 50% for F/T and reverse-phase liposomes, respectively.

### Other applications

The polymeric films obtained by F/T share the same synthesis principle as hydrogels, with modifications in the method that gives rise to thin matrices with high tensile strength and low water absorption capacity, which favors their application in the administration of drugs. Maneewattanapinyo et al. [110, 111] studied drug delivery in gelatin/PVA films loaded with 5% by weight lidocaine/aspirin and lidocaine/diclofenac, developed by the F/T method. The authors mixed the drugs in the polymer solution and obtained the transdermal patch by freezing for 8 h at -20 °C and thawing for 4 h at 25 °C for 10 cycles. The obtained film presented good stability during the 3-month study period when kept at 4 °C or room temperature, and polymer blending by F/T provided controlled drug release.

Due to their potential applications, several research groups have investigated the effect of the F/T process on the mechanical properties of films. François and Daraio prepared films from scleroglucan (Scl) hydrogels and glycerol as a plasticizer for application in drug delivery. The authors performed a drying method at room temperature and a cyclical F/T process. Films prepared through F/T exhibited a significant increase in tensile strength and decreased swelling (directly related to the number of F/T cycles) compared to films air-dried alone. Augmented cross-linking points per hydrogen bond can explain these physical differences with increasing F/T cycles [112]. On the other hand, PVA films have been prepared by water evaporation from the F/T-cycled gel. The maximum stress for the film fabricated with 10 F/T cycles was two times greater (46.2 MPa) than for a PVA film without F/T cycles (22.3 MPa). Furthermore, when the film was annealed at 130 °C, the maximum stress increased (181 MPa) compared to PVA films prepared with cross-linking chemical additives. The unannealed F/T cyclized film consisted of many small crystallites that act as a cross-linking point, but the annealed film comprised larger crystallites [113]. Another PVA-based film was obtained by removing water from a hydrogel using F/T cycles. Tensile strength and Young’s modulus increased, and elongation at break decreased when increasing F/T cycles. The tensile strength and Young’s modulus of PVA films increased up to 255 MPa after seven cycles [114]. The F/T method also allows for improved stability of films. Li et al. [115] developed polysaccharide-based sodium alginate (SA) and hyaluronic acid (HA) films and demonstrated that SA/HA film swelling and solubility decreased by half during the F/T period and that the material had the potential to overcome the poor water vapor barrier problems traditionally experienced with these films. Scanning electron microscopy revealed that the cross sections of the films are uniform and compact after two F/T treatments.

The stability of drugs, antibodies, or viral vectors has been another application of the F/T method. An example of this is the evaluation made by Bee et al. [116], in which the authors evaluated the influence of F/T cycles on the stability of adeno-associated viruses (AAV) serotypes, taking as a starting point the increase in the number of clinical studies evaluating different AAV serotypes as vectors for gene therapy. The researchers exposed AAV8 and AAV9 at low and high concentrations to five F/T cycles. The quality attributes of AAV8 and AAV9 remained within acceptable ranges, and the potency and concentration were unchanged within method variability. There was a minor increase in non-encapsulated DNA released from AAV8 after F/T in a phosphate-buffered saline formulation (PBS). The DNA release during F/T was minimized in a formulation with a low buffer concentration not detected in a sucrose formulation.

Storing drug substances at subzero temperatures mitigates potential risks associated with liquid storage, such as degradation and shipping stress, making it the best solution for long-term storage. F/T studies can provide valuable knowledge on the molecule even when performed from an early formulation image. An example of the above is monoclonal antibodies, therapeutic substances that are important to be in a stable form throughout the production process and during storage. A study conducted by Rayfield et al. [117] provided a scale-down qualification of freeze methods using several containers of varying size, subjected to five temperature control points (-20, -40, -70, and -135 ºC) and three thawing methods (controlled rate, uncontrolled rate with agitation, uncontrolled rate without agitation) on four types of monoclonal antibodies type IgG. The data obtained in this study demonstrated no impact on drug substance quality after undergoing the typical F/T cycle process for the variables evaluated. However, Kordulewska et al. [118] assessed the effects of storage duration and multiple F/T cycles on cytokine stability on serum samples stored at 4 ºC for 1–7 and 30 days and underwent multiple F/T cycles. The eight examined cytokines presented significant degradation after 4 days of sample storage at 4 ºC. They were affected by freezing at -20 ºC and thawing, and IL-1 and IL-8 exhibited significant concentration decrease after two F/T cycles. It has also been determined that cytokines in serum samples after multiple F/T cycles had better stability when stored at -80 ºC than at -20 ºC.

The construction of polymeric scaffolds for cell growth has been performed by the F/T method. Sanchez-Cardona et al. [119] prepared chitosan/gelatin/PVA scaffolds by F/T cycles followed by lyophilization for tissue engineering applications. They obtained three-dimensional lattice scaffolds with porosity higher than 80%, with heterogeneous pore sizes ranging from 0.6- 265 μm. The scaffoldings had a maximum swelling capacity greater than 600% and a controllable degradation rate, retaining their three-dimensional network structure after 28 days in PBS. The F/T technique positively influenced pore formation and scaffold size, and these features have an essential effect on nutrient diffusion, adhesion, and cell viability.

The synthesis of porous foams by F/T is also being widely explored, due to the potential applications that they may have, such as thermal insulation, gas adsorption, selective absorption of liquids for environmental remediation, and biotechnological applications [120]. Cellulose nanofibril foams are porous materials with exceptional mechanical properties resulting from the high strength-to-weight ratio of nanofibrils. Antonini et al. [120] developed an optimized manufacturing process for highly porous cellulose foams based on the F/T technique controlled at ambient pressure and obtained foams with ultra-high porosity (99.4%), a density of 10 mg/cm^3^, and liquid absorption capacity of 100 L/kg. Similarly, the use of microfibrillated cellulose has also been explored for the manufacture of foams employing the F/T method, which allows the production of mechanically stable and lightweight porous structures under low-cost environmental drying conditions [121]. Compared to other dense materials, foams have the advantages of being lightweight in structure, with a higher surface-to-weight ratio, and permeable to fluids and/or gases.

**Fig. 3:**
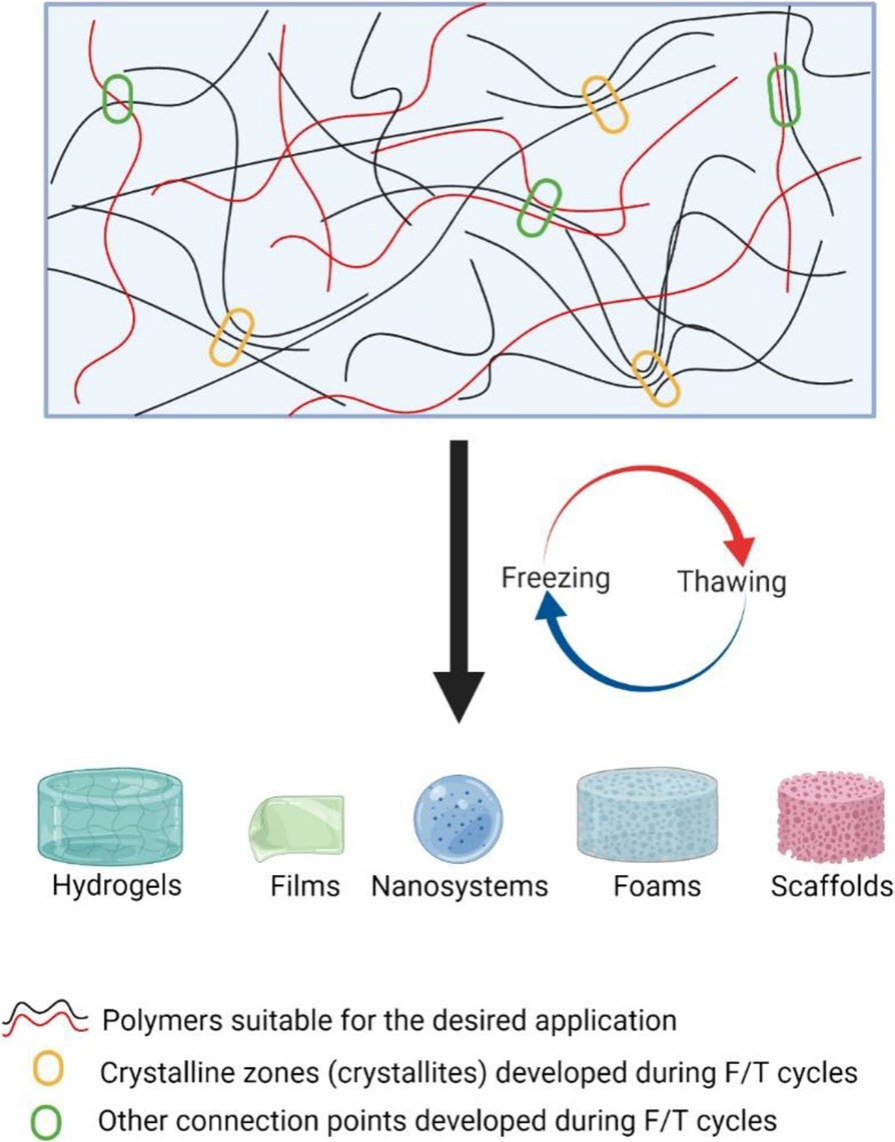
Freeze-thaw method is applied to polymeric solutions to develop matrices with potential pharmaceutical applications

As described above, applying the F/T method on polymer solutions involves forming ice crystals that promote the development of phases rich in polymers, where interactions between polymer chains are promoted (Fig. 3). During the F/T cycles, crystalline and amorphous zones are formed. It has been observed that the amorphous degree decreases when the polymer blend is subjected to more F/T cycles. This property and suitable polymers give rise to different polymeric matrices with potential pharmaceutical applications, such as hydrogels, nanosystems, foams, and films.

## Optimization of freeze/thaw conditions under the QbD approach

The quality of pharmaceutical products is entirely related to guaranteeing safety and efficacy in their use. From this, regulatory agencies worldwide have designed norms and standards to evaluate drug quality thoroughly. Unfortunately, gaps still lead to inconsistencies in meeting all requirements [122]. Quality by Design (QbD) is a pharmaceutical development approach widely used in the global pharmaceutical industry, focused on the generation of scientifically based knowledge that allows the detection of the primary sources of variation affecting the quality of the final product [123]. QbD as a systematic drug development process allows generating a robust product based on risk analysis, where, through statistical tools such as Design of Experiments (DoE), response surface methodology (RSM), regression models, and optimization techniques, the major sources of variation in the manufacturing process and formulation that determine the quality behavior are known [124, 125]. Almost at the final phase of QbD, a design space is defined, which is a region of operability where the effect of Critical Process Parameters (CPPs) and Critical Material Attributes (CMAs) on Critical Quality Attributes (CQAs) is detected and controlled, leading these variables to a state of consistency, reproducibility, and compliance with pre-established specifications [125, 126]. The CPPs and CMAs, evaluated as independent variables or factors, are fixed from the knowledge of the process and based on risk analysis to being deliberately manipulated at different levels of experimentation. At the same time, the CQAs are studied as a response or dependent variable. CQAs describe the functional quality of the product and are commonly determined following regulatory standards and/or set in a Quality Target Product Profile (QTPP), in which its criticality is determined based on the direct or indirect influence on the safety and efficacy of the product, and the severity of harm to the patients [126, 127].

A typical F/T process is usually employed during the processing and handling of drug substances and/or drug products to obtain dosage forms or enhance chemical and physical stability. However, the products are subjected to different steps or cycles that involve high physical stress that could change properties associated with quality attributes [128]. Then, product resistance to this stress must be studied to detect F/T susceptibility timely. After, DoE in a multivariate study, with multiple process parameters that are varied simultaneously, has been applied to understand how the process performs, what factors are critical, and what models are suitable to optimize response variables. The application of the DoE and QbD approach in the F/T process focuses on the impact analysis of process variables on chemical and/or physical stability. Most reported studies were mainly carried out to establish indicators of quality during the manufacture in this sense for several drug products such as proteins [129–131], nanosuspensions [132], and self-micro emulsifying delivery systems [133]. For instance, to evaluate the stability of formulations of monoclonal antibodies and nanopharmaceuticals through experimental designs, it has generated multivariate analysis models that allow predicting the impact on CQAs of cryoprotectants and stabilizing agents in the formulations (as examples of CMAs). Together with evaluating the cycles of F/T by sequential cycling between 5 to -40 °C, optimizing the concentration of excipients, the number of cycles, freeze temperatures, and the duration of the F/T stages studied as factors or CPPs [134]. In contrast, changes in purity, subvisible particles, homogeneity, pH, osmolality, particle size, aggregates, Z potential, oxidation, glycosylation, conformation, and potency have been evaluated as target product quality attributes or CQAs [132, 134, 135]. A complete QbD approach that includes clearly defined phases has been applied mainly in the development of biopharmaceuticals [135–137], in which the initial risk assessment for the formulation is carried out through a cause-and-effect analysis of all theoretical parameters supposed to have an impact on quality attributes, set as response variables during the lyophilization or F/T process [128].

Recently, the effect of F/T process variables on induced cross-linked hydrogels has been evaluated with this approach to optimize polymeric systems used for wound dressing and tissue engineering, whose rheological and mechanical properties were analyzed as CQAs [138, 139]. On the other hand, the impact of the mentioned critical parameters has also been studied with this statistical approach in other areas, such as foods, where similar CPPs and response variables have been set up [140, 141].

In the establishment of CPPs, it is crucial to keep in mind all the stages that are involved in the F/T unit operation when it is executed in the obtaining of pharmaceutical products so that, based on risk analysis, it identifies the variables that have the most significant impact on the quality attributes of the final product or the resulting intermediate product. Fabrication at the industrial or pilot scales involves vessels configured with cooling skids that circulate heat transfer fluid (HTF) through the vessel’s jacket and internal coils. For freezing, HTF temperatures are typically maintained between -55 and -45 °C, pumping the liquefied bulk at a modest flow rate (1 L/min), with thaw cycles that occur between 9 and 12 h, for tanks between 200 and 300 L [128, 142]. Generally, during the F/T process, CCPs such as the steps’ duration, the medium’s volume, HTF temperature, and the number of cycles are frequently fixed. Table 2 summarizes some CPPs, CMAs, and CQAs studied and analyzed for F/T processes.

**Table 2:** Standard variables studied under a DoE and QbD approach in the freeze/thaw process

**Product**	**Factors (CPPs) – tested levels**	**Response Variables (CQA)**	**Observations/ Main findings**	**Reference**
L-lactic dehydrogenase (LDH) in a 700-mL pilot-scale	• Freezing time (1–12 h) • Thawing time (1–12 h) • Holding time (0–11 h) • Set temperature (–10ºC, –24ºC, –38ºC) • Fill volume (250 mL, 475 mL, 700 mL) • Recirculation of the protein solution during thawing (Yes or No)	• Specific activity (%) • Protein concentration (%) • Aggregate number (10^4^/mL) • Aggregate size (μm_ECD_)	Application of A- and D-optimal experimental design. The freezing temperature was the most critical process parameter of LDH stability	[129]
Lyophilized LDH from rabbit muscle	Freeze temperature (− 40 ºC)	• LDH activity (%) • Protein concentration (%) • Aggregation (detection)	The used model helps predict temperature development and the spatial geometry of macroscopic cryoconcentration during a freezing process	[131]
Monoclonal antibody (mAb1)	• Start temperature (5, 10, 20, and 30 ºC • HTF temperature (-20, -30, -40, and -50 ºC) • Freeze-thaw cycles • Presence of cryoprotectants and surfactants	• Soluble aggregates (%) • Turbidity (NTU) • Polydispersity index by DLS (%) • Particles > 1 μm by LO (particles/mL)	Application of a Full Factorial Design. Intermediate cooling and freezing times favor quality attributes, where cryoprotectants and non-ionic surfactants in formulations reduce the effect of the freeze-thaw process on stability	[136]
Myoglobin and LHD	• Four different freeze-thaw modalities • Presence of surfactants	• Enzymatic activity (%) • changes in the conformation (UV absorbance)	Factorial design with three-way ANOVA. Model outputs suggested that a low cooling rate during freezing is beneficial for proteins prone to unfold at the ice surface In contrast, a high freezing rate improves the recovery of extremely unstable molecules in bulk	[130]
Interferon (IFN), two monoclonal antibodies (MAbs), and an Fc-fusion protein	• Freezing rate (2–10 h) • Thawing rate (2–10 h)	Aggregates content	Application of a 2^k^ Factorial Design: two-factors, two-levels face-centered composite surface response. Once the design space was created, it was possible to define the most appropriate conditions of freeze-thaw to get protein stability	[137]
Liquid Drug Nanosuspensions (Itraconazole)	• Freezing rate = − 1 (0.2◦C/min), + 1 (4◦C/min) • Steric stabilizer concentration = − 1 (20 mg/mL), + 1 (33 mg/mL) • Cryoprotectant concentration = − 1 (25 mg/mL), + 1 (50 mg/mL)	• Particle size (nm) • Nanoparticle stability	2^3^ for a complete factorial design. The study revealed the combination of “steric stabilizer concentration” and “cryoprotectant concentration” must be carefully chosen to impart nanoparticle stability during freezing	[132]
self-microemulsifying astaxanthin	Freeze‑thaw cycles (1–3)	• Droplet size (nm) • PDI • Zeta potential (mV) • Active ingredient content (%)	Optimized formulation by mixture design. Results showed stability properties when the freeze-thaw study was conducted	[133]

## Conclusions

In conclusion, the freeze-thaw (F/T) method is a valuable tool in the processing and handling of polymers to enhance their chemical and physical stability and improve formulation stability in pharmaceutical applications. However, the success of the F/T method depends on the process conditions, chosen polymers, temperature, time, and the number of cycles, which require optimization before using this method. The optimization of these conditions and variables is essential to ensure that the final product meets the required quality attributes, including stability, efficacy, and safety.

The F/T method has been used in a wide range of pharmaceutical applications, including hydrogels, emulsions, and nanosystems, among others. In hydrogels, for example, F/T has been used to prevent the need for toxic cross-linking agents and to promote a concentrated product and better stability in emulsions. However, the use of F/T in these applications is limited by their characteristics, such as porosity, flexibility, swelling capacity, drug loading, and drug release capacity, which depend on the optimization of process conditions and the kind and ratio of polymers, temperature, time, and the number of cycles.

Changes in the process variables, such as freezing conditions (temperature of freezing and the number of cycles), can alter the mechanical, chemical, drug loading, and release capacity or physical stability of pharmaceutical applications. For example, increasing the number of freezing cycles decreases the swelling degree and increases the tensile strength of hydrogels, and nanoparticle coating can also be affected. Therefore, it is crucial to optimize these process variables to obtain the desired quality attributes and prevent unexpected changes in the product’s properties.

The systematic methodology of quality by design (QbD) is an experimental approach that allows the detection of primary variation sources that affect the final product’s quality. The use of design of experiments (DoE), a tool of QbD, has been applied to understand the critical factors and what models are suitable to optimize response variables. However, further studies are necessary to generate scientific knowledge that detects the primary sources of variation affecting the final product’s quality.

Future studies could focus on the QbD pharmaceutical development approach to obtain more information about the F/T method’s optimization. In addition, the use of new polymers, combinations of polymers, and different processing conditions could also be explored to enhance the properties of pharmaceutical applications processed with F/T. Moreover, other techniques such as cryomilling and cryogrinding can be used in combination with F/T to optimize the properties of pharmaceutical applications.

Overall, the present review provides insights into the impact and effects of the F/T process on the physical, mechanical, and chemical properties of diverse pharmaceutical applications and highlights the importance of optimizing F/T conditions and variables for safe and effective use in the pharmaceutical industry. The F/T method can contribute to the development of new drug products with improved stability and efficacy, and its optimization can reduce the need for toxic cross-linking agents and improve the efficiency of drug delivery systems.

## Data Availability

Not Applicable.

## Abbreviations

TA: Acid Tannic
AAV: Adeno-Associated Viruses
W: Aqueous Phase
CMAs: Critical Material Attributes
CPPs: Critical Process Parameters
CQAs: Critical Quality Attributes
ºC: Degree Centigrade
DNA: Deoxyribonucleic Acid
DoE: Design of Experiments
DSC: Differential Scanning Calorimetry
F/T: Freeze-Thaw
HTF: Heat Transfer Fluid
HA: Hyaluronic Acid
IFN: Interferon
IL-1: Interleukin-1
IL-8: Interleukin-8
LDH: Lactate Dehydrogenase
LN2: Liquid Nitrogen
MPa: Megapascal
MAbs: Monoclonal Antibodies
NTU: Nephelometric Turbidity Unit
O: Oil Phase
PVA: Poly(vinyl alcohol)
PEG: Polyethylene glycol
PVP: Polyvinylpyrrolidone
QbD: Quality by Design
QTPP: Quality Target Product Profile
RSM: Response Surface Methodology
siRNA: Small Interfering Ribonucleic Acid
SA: Sodium Alginate
UV-VIS: Ultraviolet-Visible Spectroscopy
W/O: Water in oil Emulsion
